# Marine Bryozoa of Greece: an annotated checklist

**DOI:** 10.3897/BDJ.4.e10672

**Published:** 2016-11-01

**Authors:** Vasilis Gerovasileiou, Antonietta Rosso

**Affiliations:** ‡ Institute of Marine Biology, Biotechnology and Aquaculture, Hellenic Centre for Marine Research, Heraklion, Greece; § Sezione di Scienze della Terra, Dipartimento di Scienze Biologiche, Geologiche e Ambientali, Università di Catania, Catania, Italy; | Unità di Ricerca di Catania, Consorzio Interuniversitario per le Scienze del Mare (CoNISMa), Rome, Italy

**Keywords:** Biodiversity, Aegean Sea, Sea of Crete, Levantine Sea, Ionian Sea, Eastern Mediterranean.﻿

## Abstract

**Background:**

Until today, a complete checklist of Bryozoa of the Greek seas had never been published and species records were scattered in several taxonomic and ecological studies. The aim of this paper is to produce a first checklist of marine bryozoan species of Greece, in the framework of the Greek Taxon Information System (GTIS) initiative of the LifeWatchGreece Research Infrastructure (ESFRI), by reviewing the existing literature and following the recent trends in the taxonomy of this group. ﻿

**New information:**

The marine bryozoan fauna of Greece comprises 237 species, classified in 127 genera, 66 families, 3 orders, and 2 classes. The vast majority belongs to the class Gymnolaemata (177 Cheilostomatida and 21 Ctenostomatida), while the remaining 39 species are Stenolaemata (all Cyclostomatida). Among these species, 12 are considered endemic to the eastern Mediterranean, while another 12 species are non-indigenous.

## Introduction

The Mediterranean bryozoan fauna represents about 9.6% of the known global diversity of Bryozoa (Rosso and Di Martino 2016). However, current knowledge on the bryozoan fauna of this basin is highly biased due to differences in research effort for different taxa, marine areas and habitat types. Recent efforts in the south-eastern Mediterranean Sea have significantly increased our knowledge from areas which were previously considered “data-poor” spots for bryozoans, revealing a considerable proportion of non-indigenous taxa (e.g. Harmelin et al. 2016, Sokolover et al. 2016). Furthermore, information on Bryozoa from several Mediterranean areas is fragmented in old publications and grey literature; the collation of updated checklists (e.g. Koçak and Aydın Önen 2014) by experts could contribute to the mapping of diversity for understudied taxa and areas (Bailly et al. 2016, this special collection).

Sporadic records of bryozoans from the Aegean Sea can be found in old taxonomic studies, including the descriptions of the cheilostome species *Calpensia nobilis*, *Hippaliosina depressa* and *Watersipora cucullata* from this marine region (Busk 1854), based on material collected from shallow water macroalgae by E. Forbes. The first studies focusing on Bryozoa of the eastern Mediterranean basin, the Greek seas included, took place in the late 1960s (Harmelin 1969), based on material that was collected with dredges during the oceanographic expedition of the French research vessel “Jean Charcot” (1968). A total of 101 species belonging to all the 3 living bryozoan orders, were found, including several new records for the Mediterranean fauna. Later, the taxonomic study by Hayward (1974) on the cheilostome Bryozoa of Chios Island (north-eastern Aegean) yielded 101 species, including rare records and new species. Sharing only 47 cheilostomes with Harmelin’s list mentioned above, this study increased the eastern Mediterranean bryozoan diversity by 54 species.

More recently, few taxonomic and ecological studies have focused on the bryozoan fauna of Greece (e.g. Castritsi-Catharios and Marcopoulou-Diacantoni 1983, Castritsi-Catharios and Kiortis 1984, Castritsi-Catharios and Kiortis 1985, Castritsi-Catharios et al. 1985a, Castritsi-Catharios et al. 1985b, Castritsi-Catharios et al. 1986a, Castritsi-Catharios et al. 1986b, Castritsi-Catharios and Ganias 1989, Ganias 1990), while a considerable number of records has been included in general ecological studies examining a variety of habitats (e.g. Simboura et al. 1995, Conides et al. 1999, Morri et al. 1999, Antoniadou and Chintiroglou 2005, Gerovasileiou et al. 2013, Gerovasileiou et al. 2015). A complete checklist of Bryozoa however had never been published from Greece. The aim of the present study is to give a first annotated checklist of Bryozoa from the Greek seas.

## Materials and methods

The checklist presented in this paper was compiled in the framework of the Greek Taxon Information System (GTIS), an initiative of the LifeWatchGreece Research e-Infrastructure aiming to produce a complete inventory of the known biodiversity of Greece (Bailly et al. 2016, this special collection). As this is the first attempt to compile a checklist of bryozoan diversity of the Greek seas, we performed an exhaustive literature review of bryozoan records. All taxa were cross-checked for synonymies and dubious records against the World List of Bryozoa (recent and fossil), the World Register of Marine Species (Bock and Gordon 2016, WoRMS Editorial Board 2016), and the recent update of the Mediterranean bryozoan diversity by Rosso and Di Martino (2016). Taxa identified as cf., as well as those left in open nomenclature, and fossil records were not included in the list. The checklist is annotated with the literature references reporting the presence of each species from Greek waters, as well as with notes on the nomenclature of some taxa. Non-indigenous bryozoans are also marked (NIB). The checklist follows the alphabetical order.

## Checklists

### Checklist of Bryozoa known to occur in the Greek seas

#### Gymnolaemata

Allman, 1856

#### Cheilostomatida

Busk, 1852

#### Adeonidae

Busk, 1884

#### Adeonella

Busk, 1884

#### Adeonella calveti

(Canu & Bassler, 1930)

##### Notes

Gautier 1962

#### Adeonella pallasii

(Heller, 1867)

##### Notes

Castritsi-Catharios et al. 1985a, Castritsi-Catharios et al. 1985b, Castritsi-Catharios et al. 1986a, Ganias 1990, Rosso and Novosel 2010, Chimenz Gusso et al. 2014, Gerovasileiou et al. 2015​

#### Reptadeonella

Busk, 1884

#### Reptadeonella violacea

(Johnston, 1847)

##### Notes

Harmelin 1969, Hayward 1974, Castritsi-Catharios and Marcopoulou-Diacantoni 1983, Castritsi-Catharios and Kiortis 1984, Castritsi-Catharios et al. 1986a (as *Reptoporellina*, probably a mispelling), Ganias 1990, Morri et al. 1999, Antoniadou and Chintiroglou 2005

#### Aeteidae

Smitt, 1868

#### Aetea

Lamouroux, 1812

#### Aetea anguina

(Linnaeus, 1758)

##### Notes

Harmelin 1969, Hayward 1974, Castritsi-Catharios and Kiortis 1984, Castritsi-Catharios and Kiortis 1985, Ganias 1990

#### Aetea lepadiformis

Waters, 1906

##### Notes

Ganias 1990, Morri et al. 1999

#### Aetea sica

(Couch, 1844)

##### Notes

Harmelin 1969, Hayward 1974, Castritsi-Catharios and Marcopoulou-Diacantoni 1983, Castritsi-Catharios and Kiortis 1984, Castritsi-Catharios and Kiortis 1985, Castritsi-Catharios et al. 1985a, Castritsi-Catharios et al. 1986a, Castritsi-Catharios et al. 1986b, Castritsi-Catharios and Ganias 1989, Ganias 1990, Morri et al. 1999

#### Aetea truncata

(Landsborough, 1852)

##### Notes

Harmelin 1969, Hayward 1974, Castritsi-Catharios and Kiortis 1984, Castritsi-Catharios and Kiortis 1985, Castritsi-Catharios et al. 1985a, Castritsi-Catharios et al. 1986a, Castritsi-Catharios and Ganias 1989, Ganias 1990, Morri et al. 1999

#### Antroporidae

Vigneaux, 1949

#### Rosseliana

Jullien, 1888

#### Rosseliana rosselii

(Audouin, 1826)

##### Notes

Harmelin 1969, Ganias 1990

#### Beaniidae

Canu & Bassler, 1927

#### Beania

Johnston, 1840

#### Beania cylindrica

(Hincks, 1886)

##### Notes

​Harmelin 1969, Ganias 1990, Morri et al. 1999

#### Beania hirtissima

(Heller, 1867)

##### Notes

Harmelin 1969, Hayward 1974, Castritsi-Catharios and Marcopoulou-Diacantoni 1983, Castritsi-Catharios and Kiortis 1984, Castritsi-Catharios et al. 1986a, Castritsi-Catharios and Ganias 1989, Ganias 1990, Simboura et al. 1995, Morri et al. 1999

#### Beania magellanica

(Busk, 1852)

##### Notes

Harmelin 1969, Hayward 1974, Castritsi-Catharios and Kiortis 1984, Castritsi-Catharios and Ganias 1989, Ganias 1990

#### Beania mirabilis

Johnston, 1840

##### Notes

​Hayward 1974, Castritsi-Catharios and Ganias 1989, Ganias 1990

#### Bitectiporidae

MacGillivray, 1895

#### Metroperiella

Canu & Bassler, 1917

#### Metroperiella lepralioides

(Calvet, 1903)

##### Notes

Harmelin 1969, Hayward 1974, Ganias 1990, Morri et al. 1999

#### Pentapora

Fischer, 1807

#### Pentapora fascialis

(Pallas, 1766)

##### Notes

Ganias 1990, Antoniadou and Chintiroglou 2005, Chimenz Gusso et al. 2014

#### Schizomavella

Canu & Bassler, 1917

#### ﻿Sc﻿hiz﻿omav﻿ella rudis

﻿(Manzoni, 1869﻿)

##### Notes

In the absence of type material, the validity of *S. rudis* has been questioned by Reverter-Gil et al. (2012). Two new species, *Stephanotheca watersi* Reverter-Gil, Souto & Fernandez-Pulpeiro, 2012 and *Schizomavella adriatica* Reverter-Gil, Souto, Novosel & Tilbrook, 2016 have been erected to allocate some records from the western Basin and the Adriatic, respectively. Nevertheless, records and material from the Aegean were not considered and hence they are provisionally still reported as *S. rudis* (see also Rosso and Di Martino 2016).

#### Schizomavella (Calvetomavella) discoidea

(Busk, 1859)

##### Notes

Harmelin 1969, Hayward 1974, Morri et al. 1999; the status of these records needs to be checked (see Rosso and Di Martino 2016​).

#### Schizomavella (Schizomavella) asymetrica

(Calvet, 1927)

Schizomavella (Schizomavella) asymetrica Synonym valid for *Calyptotheca triarmata* Hayward, 1974 (see Rosso et al. 2010​).

##### Notes

Hayward 1974

#### Schizomavella (Schizomavella) cornuta

(Heller, 1867)

##### Notes

Harmelin 1969, Hayward 1974, Castritsi-Catharios and Ganias 1989, Ganias 1990, Simboura et al. 1995, Morri et al. 1999; Mediterranean colonies recorded as *S. auriculata*, *S. auriculata cuspidata* and *S. cuspidata* possibly belong to this species.

#### Schizomavella (Schizomavella) hastata

(Hincks, 1862)

##### Notes

Morri et al. 1999​

#### Schizomavella (Schizomavella) linearis

(Hassall, 1841)

##### Notes

Gautier 1962, Harmelin 1969, Hayward 1974, Castritsi-Catharios et al. 1985a, Ganias 1990

#### Schizomavella (Schizomavella) mamillata

(Hincks, 1880)

Schizomavella linearis mamillata

##### Notes

Gautier 1962, Harmelin 1969, Hayward 1974, Morri et al. 1999

#### Bryocryptellidae

Vigneaux, 1949

#### Bryocryptella

Cossmann, 1906

#### Bryocryptella tubulata

(Busk, 1861)

##### Notes

Harmelin 1969​

#### Palmiskenea

Bishop & Hayward, 1989

#### Palmiskenea gautieri

Madurell, Zabala, Domínguez-Carrió & Gili, 2013

##### Notes

Harmelin 1969, Castritsi-Catharios et al. 1985a (as Palmiskenea aff. aviculifera), Castritsi-Catharios et al. 1986a. These records have been provisionally reported as *P. gautieri* following Madurell et al. (2013) and Rosso and Di Martino (2016).

#### Palmiskenea skenei

(Ellis & Solander, 1786)

##### Notes

Ganias 1990​

#### Porella

Gray, 1848

#### Porella concinna

(Busk, 1854)

##### Notes

Harmelin 1969​

#### Porella minuta

(Norman, 1868)

##### Notes

Harmelin 1969​

#### Bugulidae

Gray, 1848

#### Bicellariella

Levinsen, 1909

#### Bicellariella ciliata

(Linnaeus, 1758)

##### Notes

Antoniadou and Chintiroglou 2005

#### Bugula

Oken, 1815

#### Bugula calathus

(Norman, 1868)

##### Notes

Hayward 1974

#### Bugula neritina

(Linnaeus, 1758)

##### Distribution

NIB

##### Notes

Castritsi-Catharios and Kiortis 1984, Castritsi-Catharios and Kiortis 1985, Castritsi-Catharios and Ganias 1989

#### Bugulina

Gray, 1848

#### Bugulina avicularia

(Linnaeus, 1758)

##### Notes

Ganias 1990

#### Bugulina fulva

(Ryland, 1960)

##### Distribution

NIB

##### Notes

Morri et al. 1999

#### Bugulina simplex

(Hincks, 1886)

##### Notes

Castritsi-Catharios and Kiortis 1984

#### Bugulina spicata

(Hincks, 1886)

##### Notes

Castritsi-Catharios and Marcopoulou-Diacantoni 1983

#### Bugulina stolonifera

(Ryland, 1960)

##### Notes

Castritsi-Catharios and Kiortis 1984, Castritsi-Catharios and Kiortis 1985, Ganias 1990, Antoniadou and Chintiroglou 2005

#### Bugulina turbinata

(Alder, 1857)

##### Notes

​Castritsi-Catharios and Kiortis 1984, Castritsi-Catharios and Kiortis 1985

#### Crisularia

Gray, 1848

#### Crisularia aperta

(Hincks, 1886)

##### Notes

Castritsi-Catharios and Marcopoulou-Diacantoni 1983

#### Crisularia plumosa

(Pallas, 1766)

##### Distribution

NIB

##### Notes

Hayward 1974

#### Crisularia serrata

(Lamarck, 1816)

##### Distribution

NIB

##### Notes

Harmelin 1969, Ganias 1990

#### Calloporidae

Norman, 1903

#### Alderina

Norman, 1903

#### Alderina imbellis

(Hincks, 1860)

##### Notes

Hayward 1974

#### Aplousina

Canu & Bassler, 1927

#### Aplousina capriensis

(Waters, 1898)

##### Notes

Hayward 1974

#### Callopora

Gray, 1848

#### Callopora dumerilii

(Audouin, 1826)

##### Notes

Hayward 1974, Ganias 1990

#### Callopora lineata

(Linnaeus, 1767)

##### Notes

Harmelin 1969, Ganias 1990

#### Copidozoum

Harmer, 1926

#### Copidozoum planum

(Hincks, 1880)

##### Notes

Hayward 1974, Castritsi-Catharios et al. 1986b, Castritsi-Catharios and Ganias 1989, Ganias 1990

#### Copidozoum tenuirostre

(Hincks, 1880)

##### Notes

Hayward 1974, Castritsi-Catharios et al. 1986a, Castritsi-Catharios and Ganias 1989, Ganias 1990

#### Corbulella

Gordon, 1984

#### Corbulella maderensis

(Waters, 1898)

##### Notes

Hayward 1974, Ganias 1990, Morri et al. 1999

#### Crassimarginatella

Canu, 1900

#### Crassimarginatella crassimarginata

(Hincks, 1880)

##### Notes

Gautier 1962

#### Crassimarginatella solidula

(Hincks, 1860)

##### Notes

Ganias 1990

#### Ellisina

Norman, 1903

#### Ellisina gautieri

Fernandez Pulpeiro & Reverter Gil, 1993

##### Notes

​Ganias 1990

#### Parellisina

Osburn, 1940

#### Parellisina curvirostris

(Hincks, 1862)

##### Notes

Harmelin 1969, Hayward 1974, Ganias 1990

#### Retevirgula

Brown, 1948

#### Retevirgula akdenizae

Chimenz, Nicoletti & Lippi Boncambi, 1997

##### Notes

Morri et al. 1999

#### Candidae

d'Orbigny, 1851

#### Caberea

Lamouroux, 1816

#### Caberea boryi

(Audouin, 1826)

##### Notes

Harmelin 1969, Hayward 1974, Ganias 1990, Morri et al. 1999, Simboura et al. 1995

#### Cradoscrupocellaria

Vieira, Spencer Jones & Winston, 2013

#### Cradoscrupocellaria bertholletii

(Audouin, 1826)

##### Notes

Hayward 1974, Ganias 1990

#### Cradoscrupocellaria macrorhyncha

(Gautier, 1962)

##### Notes

Harmelin 1969, Hayward 1974

#### Scrupocaberea

Vieira, Spencer Jones, Winston, Migotto & Marques, 2014

#### Scrupocaberea maderensis

(Busk, 1860)

##### Notes

Castritsi-Catharios et al. 1986a, Castritsi-Catharios et al. 1986b, Castritsi-Catharios and Ganias 1989, Ganias 1990

#### Scrupocellaria

Van Beneden, 1845

#### Scrupocellaria aegeensis

Harmelin, 1969

##### Notes

Harmelin 1969, Ganias 1990

#### Scrupocellaria delilii

(Audouin, 1826)

##### Notes

Harmelin 1969, Ganias 1990, Morri et al. 1999

#### Scrupocellaria incurvata

Waters, 1897

##### Notes

Harmelin 1969, Ganias 1990

#### Scrupocellaria muricata

(Lamouroux, 1816)

##### Notes

Castritsi-Catharios and Kiortis 1984, Castritsi-Catharios et al. 1986a

#### Scrupocellaria scrupea

Busk, 1852

##### Notes

Gautier 1962, Harmelin 1969, Hayward 1974, Castritsi-Catharios and Kiortis 1984, Castritsi-Catharios and Kiortis 1985, Castritsi-Catharios et al. 1986a, Ganias 1990, Simboura et al. 1995, Morri et al. 1999

#### Scrupocellaria scruposa

(Linnaeus, 1758)

##### Distribution

NIB

##### Notes

Hayward 1974, Ganias 1990, Simboura et al. 1995, Morri et al. 1999

#### Cellariidae

Fleming, 1828

#### Cellaria

Ellis & Solander, 1786

#### Cellaria fistulosa

(Linnaeus, 1758)

##### Notes

Ganias 1990

#### Cellaria normani

(Hastings, 1947)

##### Notes

Harmelin 1969

#### Cellaria salicornioides

Lamouroux, 1816

##### Notes

Hayward 1974, Castritsi-Catharios and Kiortis 1984, Castritsi-Catharios and Kiortis 1985, Ganias 1990

#### Celleporidae

Johnston, 1838

#### Buskea

Heller, 1867

#### Buskea dichotoma

(Hincks, 1862)

##### Notes

Ganias 1990

#### Buskea nitida

Heller, 1867

##### Notes

Harmelin 1969

#### Cellepora

Linnaeus, 1767

#### Cellepora posidoniae

(Hayward, 1975)

##### Notes

Ganias 1990

#### Cellepora pumicosa

(Pallas, 1766)

##### Notes

Morri et al. 1999

#### Celleporina

Gray, 1848

#### Celleporina caminata

(Waters, 1879)

##### Notes

Gautier 1962, Harmelin 1969, Hayward 1974, Castritsi-Catharios and Kiortis 1984, Castritsi-Catharios et al. 1985a, Castritsi-Catharios et al. 1986a, Ganias 1990, Morri et al. 1999

#### Celleporina hassallii

(Johnston, 1847)

##### Notes

​Castritsi-Catharios and Kiortis 1984 (as *Cellepora hassalli*), Castritsi-Catharios et al. 1985a, Castritsi-Catharios et al. 1985b, Castritsi-Catharios et al. 1986a, Ganias 1990, Simboura et al. 1995, Morri et al. 1999

#### Celleporina lucida

(Hincks, 1880)

##### Notes

Morri et al. 1999

#### Celleporina tubulosa

(Hincks, 1880)

##### Notes

Harmelin 1969, Morri et al. 1999

#### Palmicellaria

Alder, 1864

#### Palmicellaria elegans

Alder, 1864

##### Notes

Harmelin 1969, Ganias 1990

#### Turbicellepora

Ryland, 1963

#### Turbicellepora avicularis

(Hincks, 1860)

##### Notes

Ganias 1990

#### Turbicellepora camera

Hayward, 1978

##### Notes

Hayward 1978, Ganias 1990, Morri et al. 1999

#### Turbicellepora coronopus

(Wood, 1844)

##### Notes

Hayward 1974, Ganias 1990, Gerovasileiou et al. 2015

#### Turbicellepora crenulata

Hayward, 1978

##### Notes

Ganias 1990

#### Turbicellepora magnicostata

(Barroso, 1919)

##### Notes

Ganias 1990

#### Chlidoniidae

Busk, 1884

#### Chlidonia

Lamouroux, 1824

#### Chlidonia pyriformis

(Bertoloni, 1810)

##### Notes

​Harmelin 1969, Hayward 1974, Castritsi-Catharios and Kiortis 1984, Castritsi-Catharios et al. 1986a, Castritsi-Catharios et al. 1986b, Ganias 1990, Morri et al. 1999

#### Chorizoporidae

Vigneaux, 1949

#### Chorizopora

Hincks, 1879

#### Chorizopora brongniartii

(Audouin, 1826)

##### Notes

​Gautier 1962, Harmelin 1969, Hayward 1974, Castritsi-Catharios and Marcopoulou-Diacantoni 1983, Castritsi-Catharios and Kiortis 1984, Castritsi-Catharios et al. 1985a, Castritsi-Catharios et al. 1986a, Castritsi-Catharios and Ganias 1989, Ganias 1990, Simboura et al. 1995, Morri et al. 1999, Antoniadou and Chintiroglou 2005

#### Colatooeciidae

Winston, 2005

#### Trematooecia

Osburn, 1940

#### Trematooecia ligulata

Ayari & Taylor, 2008

##### Notes

Harmelin 1969 (as *Cigclisula turrita*)

#### Crepidacanthidae

Levinsen, 1909

#### Crepidacantha

Levinsen, 1909

#### Crepidacantha poissonii

(Audouin, 1826)

##### Distribution

NIB

##### Notes

Ganias 1990​

#### Cribrilinidae

Hincks, 1879

#### Collarina

Jullien, 1886

#### Collarina balzaci

(Audouin, 1826)

##### Notes

​Hayward 1974, Castritsi-Catharios and Marcopoulou-Diacantoni 1983, Castritsi-Catharios and Kiortis 1984, Castritsi-Catharios and Kiortis 1985, Castritsi-Catharios et al. 1986a, Castritsi-Catharios and Ganias 1989, Ganias 1990

#### Figularia

Jullien, 1886

#### Figularia figularis

(Johnston, 1847)

##### Notes

​Harmelin 1969, Hayward 1974, Ganias 1990

#### Puellina

Jullien, 1886

#### Puellina (Cribrilaria) innominata

(Couch, 1844)

##### Notes

Harmelin 1969, Hayward 1974, Castritsi-Catharios et al. 1986a, Castritsi-Catharios et al. 1986b, Castritsi-Catharios and Ganias 1989, Ganias 1990

#### Puellina (Cribrilaria) hincksi

(Friedl, 1917)

##### Notes

Gautier 1962, Morri et al. 1999

#### Puellina (Cribrilaria) radiata

(Moll, 1803)

##### Notes

​Harmelin 1969, Hayward 1974, Castritsi-Catharios and Marcopoulou-Diacantoni 1983, Castritsi-Catharios and Kiortis 1984, Castritsi-Catharios and Kiortis 1985, Castritsi-Catharios et al. 1986a, Castritsi-Catharios and Ganias 1989, Ganias 1990, Simboura et al. 1995, Morri et al. 1999

#### Puellina (Cribrilaria) venusta

(Canu & Bassler, 1925)

##### Notes

Ganias 1990

#### Puellina (Glabrilaria) orientalis

Harmelin & Aristegui, 1987

##### Notes

​Morri et al. 1999

#### Puellina (Glabrilaria) pedunculata

(Gautier, 1956)

##### Notes

Harmelin 1969, Hayward 1974

#### Puellina (Puellina) gattyae

(Landsborough, 1852)

##### Notes

​Gautier 1962, Hayward 1974, Castritsi-Catharios and Marcopoulou-Diacantoni 1983, Castritsi-Catharios and Kiortis 1984, Castritsi-Catharios et al. 1986a, Castritsi-Catharios et al. 1986b, Castritsi-Catharios and Ganias 1989, Ganias 1990, Morri et al. 1999

#### Puellina (Puellina) setosa

(Waters, 1899)

##### Notes

Harmelin 1969, Ganias 1990, Morri et al. 1999

#### Cryptosulidae

Vigneaux, 1949

#### Cryptosula

Canu & Bassler, 1925

#### Cryptosula pallasiana

(Moll, 1803)

##### Notes

Hayward 1974, Castritsi-Catharios and Marcopoulou-Diacantoni 1983, Castritsi-Catharios et al. 1985a, Castritsi-Catharios et al. 1986a, Castritsi-Catharios et al. 1986b, Ganias 1990, Antoniadou and Chintiroglou 2005

#### Electridae

d'Orbigny, 1851

#### Conopeum

Gray, 1848

#### Conopeum seurati

(Canu, 1928)

##### Notes

Ganias 1990

#### Electra

Lamouroux, 1816

#### Electra pilosa

(Linnaeus, 1767)

##### Notes

Castritsi-Catharios et al. 1986a, Castritsi-Catharios and Ganias 1989, Ganias 1990, Antoniadou and Chintiroglou 2005

#### Electra posidoniae

Gautier, 1954

##### Notes

Hayward 1974, Castritsi-Catharios and Marcopoulou-Diacantoni 1983, Castritsi-Catharios and Kiortis 1984, Castritsi-Catharios and Kiortis 1985, Castritsi-Catharios et al. 1985a, Castritsi-Catharios et al. 1986a, Castritsi-Catharios and Ganias 1989, Ganias 1990

#### Epistomiidae

Gregory, 1893

#### Synnotum

Pieper, 1881

#### Synnotum aegyptiacum

(Audouin, 1826)

##### Notes

​Harmelin 1969, Hayward 1974, Ganias 1990

#### Escharinidae

Tilbrook, 2006

#### Escharina

Milne Edwards, 1836

#### Escharina dutertrei protecta

Zabala, Maluquer & Harmelin, 1993

##### Notes

​Harmelin 1969, Hayward 1974, Morri et al. 1999

#### Escharina johnstoni

(Quelch, 1884)

##### Notes

Ganias 1990

#### Escharina vulgaris

(Moll, 1803)

##### Notes

Harmelin 1969, Hayward 1974, Castritsi-Catharios et al. 1986a (as *Schizoporella vulgaris*), Castritsi-Catharios et al. 1986b (as *S. vulgaris*), Ganias 1990, ​Morri et al. 1999

#### Herentia

Gray, 1848

#### Herentia hyndmanni

(Johnston, 1847)

##### Notes

Harmelin 1969, Morri et al. 1999

#### Hippomenella

Canu & Bassler, 1917

#### Hippomenella mucronelliformis

(Waters, 1899)

##### Notes

Harmelin 1969, Hayward 1974, Ganias 1990

#### Therenia

David & Pouyet, 1978

#### Therenia rosei

Berning, Tilbrook & Rosso, 2008

##### Notes

​Hayward 1974 (as *Escharina porosa*), Ganias 1990 (as *E. porosa*), Berning et al. 2008

#### Exechonellidae

Harmer, 1957

#### Anarthropora

Smitt, 1868

#### Anarthropora monodon

(Busk, 1860)

##### Notes

﻿﻿Harmelin 1969, Hayward 1974, Ganias 1990, Morri et al. 1999

#### Exechonella

Duvergier, 1924

#### Exechonella antillea

(Osburn, 1927)

##### Distribution

NIB

##### Notes

Hayward 1974

#### Exochellidae

Bassler, 1935

#### Escharoides

Milne Edwards, 1836

#### Escharoides ﻿coccinea﻿

(Abildgaard, 1806)

##### Notes

Harmelin 1969, Hayward 1974, Castritsi-Catharios and Marcopoulou-Diacantoni 1983, Castritsi-Catharios and Kiortis 1984 (as *Cellepora coccinea*), Castritsi-Catharios et al. 1986a, Castritsi-Catharios and Ganias 1989, Ganias 1990, Morri et al. 1999​, Antoniadou and Chintiroglou 2005

#### Flustridae

Fleming, 1828

#### Chartella

Gray, 1848

#### Chartella papyrea

(Pallas, 1766)

##### Notes

Hayward 1974

#### Gregarinidra

Barroso, 1949

#### Gregarinidra gregaria

(Heller, 1867)

##### Notes

​Harmelin 1969, Hayward 1974, Castritsi-Catharios and Kiortis 1984

#### Hincksina

Norman, 1903

#### Hincksina flustroides

(Hincks, 1877)

##### Notes

​Hayward 1974, Ganias 1990, Morri et al. 1999

#### Haplopomidae

Gordon in De Blauwe, 2009

#### Haplopoma

Levinsen, 1909

#### Haplopoma impressum

(Audouin, 1826)

##### Notes

​Harmelin 1969, Hayward 1974, Ganias 1990, Morri et al. 1999

#### Hippaliosinidae

Winston, 2005

#### Hippaliosina

Canu, 1919

#### Hippaliosina depressa

(Busk, 1854)

##### Notes

Busk 1854, Harmelin 1969, Hayward 1974, Castritsi-Catharios and Marcopoulou-Diacantoni 1983, Castritsi-Catharios et al. 1985a, Castritsi-Catharios et al. 1985b, Castritsi-Catharios et al. 1986a, Castritsi-Catharios and Ganias 1989, Ganias 1990, Morri et al. 1999, Gerovasileiou et al. 2013, Gerovasileiou et al. 2015

#### Hippopodinidae

Levinsen, 1909

#### Hippopodina

Levinsen, 1909

#### Hippopodina ambita

(Hayward, 1974)

##### Notes

​Harmelin 1969 (as *Cosciniopsis* sp.), Hayward 1974, Ganias 1990

#### Hippopodina feegeensis

(Busk, 1884)

##### Distribution

NIB

##### Notes

Morri et al. 1999

#### Hippoporidridae

Vigneaux, 1949

#### Hagiosynodos

Bishop & Hayward, 1989

#### Hagiosynodos latus

(Busk, 1856)

##### Notes

​Hayward 1974, Ganias 1990, Morri et al. 1999

#### Hippoporidra

Canu & Bassler, 1927

#### Hippoporidra picardi

Gautier, 1962

##### Notes

Gautier 1962, Morri et al. 1999

#### Hippothoidae

Busk, 1859

#### Hippothoa

Lamouroux, 1821

#### Hippothoa flagellum

Manzoni, 1870

##### Notes

Ganias 1990

#### Lacernidae

Jullien, 1888

#### Arthropoma

Levinsen, 1909

#### Arthropoma cecilii

(Audouin, 1826)

##### Notes

Hayward 1974, Ganias 1990

#### Lanceoporidae

Harmer, 1957

#### Calyptotheca

Harmer, 1957

#### Calyptotheca rugosa

Hayward, 1974

##### Notes

Hayward 1974, Ganias 1990

#### Margarettidae

Harmer, 1957

#### Margaretta

Gray, 1843

#### Margaretta cereoides

(Ellis & Solander, 1786)

##### Notes

Harmelin 1969, Hayward 1974, Castritsi-Catharios et al. 1985a, Ganias 1990

#### Microporellidae

Hincks, 1879

#### Diporula

Hincks, 1879

#### Diporula verrucosa

(Peach, 1868)

##### Notes

Harmelin 1969, Ganias 1990

#### Fenestrulina

Jullien, 1888

#### Fenestrulina malusii

(Audouin, 1826)

##### Notes

Harmelin 1969, Hayward 1974, Castritsi-Catharios et al. 1986b, Castritsi-Catharios and Ganias 1989, Ganias 1990, Morri et al. 1999

#### Microporella

Hincks, 1877

#### Microporella coronata

(Audouin, 1826)

##### Distribution

NIB

##### Notes

Harmelin et al. (2011) suggested that specimens from the Aegean Sea reported as *Microporella orientalis* could belong to *M. coronata* (followed by Rosso and Di Martino 2016). Recorded by Hayward 1974, Morri et al. 1999.

#### Microporella ciliata

(Pallas, 1766)

##### Notes

This taxon corresponds to a complex of species (Rosso et al. 2010, Rosso and Di Martino 2016). Recorded by Harmelin 1969, Castritsi-Catharios and Marcopoulou-Diacantoni 1983, Castritsi-Catharios et al. 1986a, Ganias 1990, Antoniadou and Chintiroglou 2005.

#### Microporella marsupiata

(Busk, 1860)

##### Notes

Hayward 1974

#### Microporidae

Gray, 1848

#### Calpensia

Jullien, 1888

#### Calpensia nobilis

(Esper, 1796)

##### Notes

Busk 1854, Hayward 1974, Castritsi-Catharios and Marcopoulou-Diacantoni 1983, Castritsi-Catharios and Kiortis 1984, Castritsi-Catharios and Kiortis 1985, Castritsi-Catharios and Ganias 1989, Ganias 1990, Morri et al. 1999

#### Coronellina

Prenant & Bobin, 1966

#### Coronellina fagei

(Gautier, 1962)

##### Notes

Harmelin 1969, Hayward 1974

#### Micropora

Gray, 1848

#### Micropora coriacea

(Johnston, 1847)

##### Notes

Hayward 1974, Ganias 1990

#### Mollia

Lamouroux, 1821

#### Mollia multijuncta

(Waters, 1879)

##### Notes

Hayward 1974, Morri et al. 1999

#### Mollia patellaria

(Moll, 1803)

##### Notes

Gautier 1962, Harmelin 1969, Hayward 1974, Ganias 1990, Morri et al. 1999

#### Monoporellidae

Hincks, 1882

#### Monoporella

Hincks, 1881

#### Monoporella bouchardii

(Audouin, 1826)

##### Notes

Mediterranean specimens of *Monoporella nodulifera* and *M. fimbriata carinifera* actually belong to this species, as suggested by Harmelin (2014a) and followed by Rosso and Di Martino (2016). Recorded by Harmelin 1969, Hayward 1974, Ganias 1990, Morri et al. 1999.

#### Myriaporidae

Gray, 1841

#### Myriapora

de Blainville, 1830

#### Myriapora truncata

(Pallas, 1766)

##### Notes

​Harmelin 1969, Hayward 1974, Morri et al. 1999, Gerovasileiou et al. 2015

#### Onychocellidae

Jullien, 1882

#### Onychocella

Jullien, 1882

#### Onychocella angulosa

(Reuss, 1847)

##### Notes

Hayward 1974, Ganias 1990

#### Onychocella marioni

(Jullien, 1881)

##### Notes

​Harmelin 1969, Morri et al. 1999, Gerovasileiou et al. 2015

#### Onychocella vibraculifera

Neviani, 1895

##### Notes

​Hayward 1974, Castritsi-Catharios and Marcopoulou-Diacantoni 1983, Ganias 1990

#### Smittipora

Jullien, 1882

#### Smittipora disjuncta

(Canu & Bassler, 1930)

Smittipora is a synonym of *Rectonychocella* (see Rosso and Di Martino 2016​).

##### Notes

Harmelin 1969, Hayward 1974, Ganias 1990

#### Phidoloporidae

Gabb & Horn, 1862

#### Dentiporella

Barroso, 1926

#### Dentiporella sardonica

(Waters, 1879)

##### Notes

Gautier 1962, Harmelin 1969

#### Plesiocleidochasma

Soule, Soule & Chaney, 1991

#### Plesiocleidochasma mediterraneum

Chimenz-Gusso & Soule, 2003

##### Notes

Harmelin 1969 (as *Cleidochasma* sp.), Hayward 1974 (as *Cleidochasma porcellanum*)

#### Reteporella

Busk, 1884

#### Reteporella complanata

(Waters, 1894)

##### Notes

Ganias 1990

#### Reteporella couchii

(Hincks, 1878)

##### Notes

Harmelin 1969, Ganias 1990

#### Reteporella grimaldii

(Jullien & Calvet, 1903)

Reteporella grimaldii Synonym valid for *R. septentrionalis* (see Rosso et al. 2010​).

##### Notes

​Hayward 1974, Ganias 1990, Morri et al. 1999, Antoniadou and Chintiroglou 2005, Chimenz Gusso et al. 2014

#### Reteporella mediterranea

(Hass, 1948)

##### Notes

Hayward 1974, Ganias 1990

#### Reteporellina

Harmer, 1933

#### Reteporellina delicatula

Hayward, 1974

##### Notes

Hayward 1974, Harmelin 1976, Ganias 1990

#### Rhynchozoon

Hincks, 1895

#### Rhynchozoon bispinosum

(Johnston, 1847)

##### Notes

Hayward 1974, Ganias 1990

#### Rhynchozoon neapolitanum

Gautier, 1962

##### Notes

​Harmelin 1969, Hayward 1974, Ganias 1990, Morri et al. 1999, Gerovasileiou et al. 2015

#### Rhynchozoon pseudodigitatum

Zabala & Maluquer, 1988

##### Notes

Morri et al. 1999

#### ﻿Rhy﻿nchozoon sp.﻿ ﻿1

sen﻿su Hayward, 1974

##### Notes

Hayward 1974

#### Schizoretepora

Gregory, 1893

#### Schizoretepora imperati

(Busk, 1884)

##### Notes

Chimenz Gusso et al. 2014

#### Schizoretepora serratimargo

(Hincks, 1886)

##### Notes

Gautier 1962, Harmelin 1969, Ganias 1990

#### Schizoretepora solanderia

(Risso, 1826)

##### Notes

Harmelin 1969, Ganias 1990

#### Schizotheca

Hincks, 1877

#### Schizotheca fissa

(Busk, 1856)

##### Notes

Harmelin 1969, Hayward 1974, Ganias 1990

#### Stephanollona

Duvergier, 1921

#### Stephanollona armata

(Hincks, 1861)

##### Notes

Harmelin 1969, Hayward 1974, Castritsi-Catharios and Marcopoulou-Diacantoni 1983, Castritsi-Catharios et al. 1985a, Castritsi-Catharios et al. 1985b, Ganias 1990

#### Phoceanidae

Vigneaux, 1949

#### Phoceana

Jullien in Jullien & Calvet, 1903

#### Phoceana tubulifera

(Heller, 1867)

##### Notes

Harmelin 1969, Hayward 1974, Ganias 1990

#### Romancheinidae

Jullien, 1888

#### Escharella

Gray, 1848

#### Escharella rylandi

Geraci, 1974

##### Notes

Ganias 1990

#### Hemicyclopora

Norman, 1894

#### Hemicyclopora multispinata

(Busk, 1861)

##### Notes

Ganias 1990, Morri et al. 1999

#### Hippopleurifera

Canu & Bassler, 1925

#### Hippopleurifera pulchra

(Manzoni, 1870)

##### Notes

Harmelin 1969

#### Savignyellidae

Levinsen, 1909

#### Savignyella

Levinsen, 1909

#### Savignyella lafontii

(Audouin, 1826)

##### Notes

Gautier 1962, Hayward 1974, Morri et al. 1999

#### Schizoporellidae

Jullien, 1883

#### Schizobrachiella

Canu & Bassler, 1920

#### Schizobrachiella sanguinea

(Norman, 1868)

##### Notes

Gautier 1962, Harmelin 1969, Ganias 1990, Morri et al. 1999

#### Schizoporella

Hincks, 1877

#### Schizoporella dunkeri

(Reuss, 1848)

##### Notes

Harmelin 1969, Hayward 1974, Castritsi-Catharios and Marcopoulou-Diacantoni 1983, Castritsi-Catharios et al. 1986a, Morri et al. 1999, Ganias 1990

#### Schizoporella errata

(Waters, 1878)

##### Notes

Hayward 1974, Castritsi-Catharios et al. 1985a, Ganias 1990

#### Schizoporella magnifica

Hincks, 1886

##### Notes

Harmelin 1969, Hayward 1974, Ganias 1990

#### Schizoporella unicornis

(Johnston in Wood, 1844)

##### Notes

Castritsi-Catharios et al. 1986a, Ganias 1990, Morri et al. 1999, Antoniadou and Chintiroglou 2005

#### Setosellidae

Levinsen, 1909

#### Setosella

Hincks, 1877

#### Setosella vulnerata

(Busk, 1860)

##### Notes

Hayward 1974, Ganias 1990

#### Smittinidae

Levinsen, 1909

#### Parasmittina

Osburn, 1952

#### Parasmittina raigii

(Audouin, 1826)

##### Distribution

NIB

##### Notes

Ganias 1990

#### Parasmittina rouvillei

(Calvet, 1902)

##### Notes

Harmelin 1969, Hayward 1974, Ganias 1990

#### Prenantia

Gautier, 1962

#### Prenantia cheilostoma

(Manzoni, 1869)

##### Notes

Hayward 1974, Ganias 1990

#### Prenantia inerma

(Calvet, 1906)

##### Notes

Harmelin 1969

#### Smittina

Norman, 1903

#### Smittina cervicornis

(Pallas, 1766)

##### Notes

​Gautier 1962 (as *Porella cervicornis*), Ganias 1990 (as *P. cervicornis*), Morri et al. 1999, Chimenz Gusso et al. 2014

#### Smittina colleti

(Jullien & Calvet, 1903)

##### Notes

Harmelin 1969, Ganias 1990

#### Smittina landsborovii

(Johnston, 1847)

##### Notes

Ganias 1990, Antoniadou and Chintiroglou 2005

#### Smittina remotorostrata

(Canu & Bassler, 1928)

##### Notes

Hayward 1974

#### Smittoidea

Osburn, 1952

#### Smittoidea marmorea

(Hincks, 1877)

##### Notes

Hayward 1974, Ganias 1990

#### Smittoidea ophidiana

(Waters, 1879)

##### Notes

Harmelin 1969, Hayward 1974

#### Smittoidea reticulata

(MacGillivray, 1842)

##### Notes

Gautier 1962, Morri et al. 1999, Antoniadou and Chintiroglou 2005

#### Tessaradomidae

Jullien, 1903

#### Tessaradoma

Norman, 1869

#### Tessaradoma boreale

(Busk, 1860)

##### Notes

Ganias 1990

#### Trypostegidae

Gordon, Tilbrook & Winston, 2005

#### Trypostega

Levinsen, 1909

#### Trypostega venusta

(Norman, 1864)

##### Notes

Ganias 1990

#### Umbonulidae

Canu, 1904

#### Umbonula

Hincks, 1880

#### Umbonula ovicellata

Hastings, 1944

##### Notes

Hayward 1974, Castritsi-Catharios and Kiortis 1984, Castritsi-Catharios et al. 1986a, Ganias 1990, Morri et al. 1999

#### Watersiporidae

Vigneaux, 1949

#### Watersipora

Neviani, 1896

#### Watersipora complanata

(Norman, 1864)

##### Notes

Ganias 1990

#### Watersipora cucullata

(Busk, 1854)

##### Notes

Busk 1854, Hayward 1974, Castritsi-Catharios et al. 1986b, Castritsi-Catharios and Ganias 1989, Ganias 1990. Records of *Watersipora subovoidea* (d'Orbigny, 1852) probably belong to *W. cucullata*.

#### Ctenostomatida

Busk, 1852

#### Alcyonidiidae

Johnston, 1838

#### Alcyonidium

Lamouroux, 1813

#### Alcyonidium cellarioides

(Calvet, 1900)

##### Notes

Castritsi-Catharios and Kiortis 1984, Castritsi-Catharios and Kiortis 1985

#### Alcyonidium duplex

Prouho, 1892

##### Notes

Ganias 1990

#### Alcyonidium polyoum

(Hassall, 1841)

##### Notes

Ganias 1990

#### Buskiidae

Hincks, 1880

#### Buskia

Alder, 1857

#### Buskia nitens

Alder, 1857

##### Notes

​Castritsi-Catharios and Kiortis 1984, Castritsi-Catharios and Kiortis 1985, Ganias 1990

#### Farrellidae

d'Hondt, 1983

#### Farrella

Ehrenberg, 1838

#### Farrella repens

(Farre, 1837)

##### Notes

Castritsi-Catharios et al. 1986a, Castritsi-Catharios et al. 1986b

#### Mimosellidae

Hincks, 1877

#### Bantariella

Jebram, 1973

#### Bantariella verticillata

(Heller, 1867)

##### Notes

​Castritsi-Catharios and Marcopoulou-Diacantoni 1983, Castritsi-Catharios and Kiortis 1984, Castritsi-Catharios and Kiortis 1985, Castritsi-Catharios et al. 1985a, Castritsi-Catharios et al. 1986a, Castritsi-Catharios et al. 1986b, Castritsi-Catharios and Ganias 1989, Ganias 1990, Morri et al. 1999

#### Mimosella

Hincks, 1851

#### Mimosella gracilis

Hincks, 1851

##### Notes

Harmelin 1969, Castritsi-Catharios and Kiortis 1984, Castritsi-Catharios and Kiortis 1985, Castritsi-Catharios et al. 1985a, Castritsi-Catharios et al. 1986a, Castritsi-Catharios and Ganias 1989, Ganias 1990, Morri et al. 1999

#### Nolellidae

Harmer, 1915

#### Nolella

Gosse, 1851

#### Nolella dilatata

(Hincks, 1860)

##### Notes

Harmelin 1969, Ganias 1990

#### Nolella stipata

Gosse, 1855

##### Notes

Castritsi-Catharios and Kiortis 1984, Castritsi-Catharios et al. 1986a, Castritsi-Catharios and Ganias 1989, Ganias 1990, Morri et al. 1999

#### Pherusellidae

Osburn & Soule, 1953

#### Pherusella

Soule, 1951

#### Pherusella tubulosa

(Ellis & Solander, 1786)

##### Notes

Castritsi-Catharios and Kiortis 1984, Castritsi-Catharios and Kiortis 1985, Castritsi-Catharios et al. 1986a, Ganias 1990, Morri et al. 1999

#### Vesiculariidae

Hincks, 1880

#### Amathia

Lamouroux, 1812

#### Amathia gracilis

(Leidy, 1855)

##### Notes

Ganias 1990, Morri et al. 1999

#### Amathia gracillima

(Hincks, 1877)

##### Distribution

NIB

##### Notes

Castritsi-Catharios and Kiortis 1984, Castritsi-Catharios and Kiortis 1985, Ganias 1990

#### Amathia imbricata

(Adams, 1798)

##### Notes

Castritsi-Catharios and Kiortis 1984, Castritsi-Catharios and Kiortis 1985, Castritsi-Catharios et al. 1986a, Castritsi-Catharios et al. 1986b, Castritsi-Catharios and Ganias 1989, Ganias 1990

#### Amathia lendigera

(Linnaeus, 1758)

##### Notes

Castritsi-Catharios and Ganias 1989, Ganias 1990, Morri et al. 1999

#### Amathia pruvoti

Calvet, 1911

##### Notes

Castritsi-Catharios and Marcopoulou-Diacantoni 1983, Castritsi-Catharios and Kiortis 1984, Castritsi-Catharios and Kiortis 1985, Castritsi-Catharios et al. 1986a, Castritsi-Catharios and Ganias 1989, Ganias 1990, Morri et al. 1999

#### Amathia pustulosa

(Ellis & Solander, 1786)

##### Notes

Castritsi-Catharios et al. 1986a, Castritsi-Catharios et al. 1986b

#### Amathia semiconvoluta

Lamouroux, 1824

##### Notes

Castritsi-Catharios and Ganias 1989, Ganias 1990

#### Amathia verticillata

(Delle Chiaje, 1822)

##### Distribution

NIB

##### Notes

Castritsi-Catharios and Kiortis 1984, Castritsi-Catharios and Kiortis 1985, Castritsi-Catharios et al. 1986a, Castritsi-Catharios et al. 1986b, Castritsi-Catharios and Ganias 1989, Ganias 1990, Conides et al. 1999

#### Victorellidae

Hincks, 1880

#### Victorella

Saville-Kent, 1870

#### Victorella pavida

Saville-Kent, 1870

##### Notes

Castritsi-Catharios et al. 1986a, Castritsi-Catharios et al. 1986b

#### Walkeriidae

Hincks, 1880

#### Walkeria

Fleming, 1823

#### Walkeria tuberosa

Heller, 1867

##### Notes

Castritsi-Catharios and Kiortis 1984, Castritsi-Catharios et al. 1985a, Castritsi-Catharios et al. 1986a, Castritsi-Catharios and Ganias 1989, Ganias 1990, Morri et al. 1999

#### Walkeria uva

(Linnaeus, 1758)

##### Notes

Castritsi-Catharios and Kiortis 1984, Castritsi-Catharios et al. 1986a, Castritsi-Catharios and Ganias 1989, Ganias 1990

#### Stenolaemata

Borg, 1926

#### Cyclostomatida

Busk, 1852

#### Annectocymidae

Hayward & Ryland, 1985

#### Annectocyma

Hayward & Ryland, 1985

#### Annectocyma arcuata

(Harmelin, 1976)

##### Notes

Ganias 1990

#### Annectocyma major

(Johnston, 1847)

##### Notes

​Castritsi-Catharios et al. 1985a (as *Stomatopora major*), Castritsi-Catharios et al. 1986a, Castritsi-Catharios et al. 1986b, Castritsi-Catharios and Ganias 1989 (as *S. major*), Ganias 1990, Simboura et al. 1995, Morri et al. 1999

#### Annectocyma tubulosa

(Busk, 1875)

##### Notes

Ganias 1990

#### Entalophoroecia

Harmelin, 1976

#### Entalophoroecia deflexa

(Couch, 1844)

##### Notes

Castritsi-Catharios et al. 1986a, Castritsi-Catharios et al. 1986b, Castritsi-Catharios and Ganias 1989, Ganias 1990

#### Entalophoroecia gracilis

Harmelin, 1976

##### Notes

Ganias 1990

#### Entalophoroecia robusta

Harmelin, 1976

##### Notes

Ganias 1990

#### Crisiidae

Johnston, 1838

#### Crisia

Lamouroux, 1812

#### Crisia denticulata

(Lamarck, 1816)

##### Notes

Ganias 1990

#### Crisia eburnea

(Linnaeus, 1758)

##### Notes

Castritsi-Catharios and Kiortis 1984, Castritsi-Catharios et al. 1986a, Castritsi-Catharios et al. 1986b, Castritsi-Catharios and Ganias 1989, Ganias 1990

#### Crisia elongata

Milne Edwards, 1838

##### Notes

Castritsi-Catharios et al. 1986a, Castritsi-Catharios et al. 1986b

#### Crisia fistulosa

(Heller, 1867)

##### Notes

Castritsi-Catharios and Marcopoulou-Diacantoni 1983, Castritsi-Catharios et al. 1986a, Ganias 1990

#### Crisia pyrula

Harmelin, 1990

##### Notes

Harmelin 1969 (as *Crisia* sp. II), Ganias 1990​

#### Crisia ramosa

Harmer, 1891

##### Notes

Castritsi-Catharios and Marcopoulou-Diacantoni 1983, Castritsi-Catharios et al. 1986a, Castritsi-Catharios and Ganias 1989, Ganias 1990, Simboura et al. 1995

#### Crisia sigmoidea

Waters, 1916

##### Notes

Harmelin 1969, Castritsi-Catharios et al. 1986a, Castritsi-Catharios and Ganias 1989, Ganias 1990, Simboura et al. 1995

#### Entalophoridae

Reuss, 1869

#### Mecynoecia

Canu, 1918

#### Mecynoecia delicatula

(Busk, 1875)

##### Notes

Ganias 1990

#### Frondiporidae

Busk, 1875

#### Frondipora

Link, 1807

#### Frondipora verrucosa

(Lamouroux, 1821)

##### Notes

Harmelin 1969 (as *Frondipora gracilis*), Ganias 1990, Gerovasileiou et al. 2015

#### Horneridae

Smitt, 1867

#### Hornera

Lamouroux, 1821

#### Hornera frondiculata

Lamouroux, 1821

##### Notes

Harmelin 1969, Ganias 1990, Simboura et al. 1995

#### Lichenoporidae

Smitt, 1867

#### Disporella

Gray, 1848

#### Disporella hispida

(Fleming, 1828)

##### Notes

Rosso and Di Martino (2016) maintained this taxon but it is possible that these records actually belong to a different species, *D. alboranensis* Alvarez, 1992; all records need to be validated. Recorded by Harmelin 1969, Castritsi-Catharios and Marcopoulou-Diacantoni 1983, Castritsi-Catharios et al. 1986a, Castritsi-Catharios and Ganias 1989 (as *Lichenopora hispida*), Ganias 1990, Simboura et al. 1995, Morri et al. 1999.

#### Patinella

Gray, 1848

#### Patinella radiata

(Audouin, 1826)

##### Notes

​Castritsi-Catharios and Marcopoulou-Diacantoni 1983, Castritsi-Catharios and Kiortis 1984, Castritsi-Catharios et al. 1985a, Castritsi-Catharios et al. 1986a, Castritsi-Catharios and Ganias 1989, Ganias 1990, Simboura et al. 1995, Morri et al. 1999, Antoniadou and Chintiroglou 2005

#### Patinella verrucaria

(Linnaeus, 1758)

##### Notes

Castritsi-Catharios et al. 1986a, Castritsi-Catharios and Ganias 1989, Ganias 1990

#### Oncousoeciidae

Canu, 1918

#### ﻿'Microecia'

Canu, 1918

#### ﻿'M﻿icroecia' occulta

(Harmelin, 1976)

﻿'M﻿icroecia' occulta See Rosso et al. (2010)

##### Notes

Ganias 1990

#### Plagioeciidae

Canu, 1918

#### Cardioecia

Canu & Bassler, 1922

#### Cardioecia watersi

(O'Donoghue & de Watteville, 1939)

##### Notes

Harmelin 1969 (as *Entalophora rugosa*), Harmelin 1976, Castritsi-Catharios et al. 1986b, Castritsi-Catharios and Ganias 1989, Ganias 1990

#### Desmeplagioecia

Canu & Bassler, 1920

#### Desmeplagioecia amphorae

Harmelin, 1976

##### Notes

Ganias 1990

#### Desmeplagioecia violacea

Harmelin, 1976

##### Notes

Ganias 1990

#### Diplosolen

Canu, 1918

#### Diplosolen obelius

(Johnston, 1838)

Diplosolen obelius The use of the name *D. obelius* for this species has been proposed by Rosso and Di Martino (2016) in spite of '*obelium*' and '*obelia*', used inconsistently in the literature to ensure accordance to the gender of the genus name.

##### Notes

Harmelin 1969, Ganias 1990, Morri et al. 1999

#### Plagioecia

Canu, 1918

#### Plagioecia dorsalis

(Waters, 1879)

##### Notes

Ganias 1990

#### Plagioecia inoedificata

(Jullien, 1882)

##### Notes

Ganias 1990

#### Plagioecia patina

(Lamarck, 1816)

##### Notes

Harmelin 1969, Ganias 1990, Antoniadou and Chintiroglou 2005

#### Plagioecia platydiscus

Harmelin, 1976

##### Notes

Ganias 1990

#### Plagioecia sarniensis

(Norman, 1864)

##### Notes

Harmelin 1976, Ganias 1990

#### Terviidae

Canu & Bassler, 1920

#### Tervia

Jullien, 1883

#### Tervia irregularis

(Meneghini, 1844)

##### Notes

Ganias 1990

#### Tubuliporidae

Johnston, 1838

#### Harmelinopora

Brood, 1976

#### Harmelinopora indistincta

(Canu and Bassler, 1929)

Harmelinopora indistincta See Rosso and Di Martino (2016) and references therein

##### Notes

Castritsi-Catharios and Marcopoulou-Diacantoni 1983, Ganias 1990

#### Idmidronea

Canu & Bassler, 1920

#### Idmidronea coerulea

Harmelin, 1976

Idmidronea coerulea See Rosso et al. (2010) and references therein

##### Notes

Ganias 1990

#### Idmidronea triforis

(Heller, 1867)

Idmidronea triforis See Rosso et al. (2010) and references therein

##### Notes

Harmelin 1969 (as *Idmidronea atlantica*), Ganias 1990 (as *I. atlantica*)

#### Platonea

Canu & Bassler, 1920

#### Platonea stoechas

Harmelin, 1976

##### Notes

Harmelin 1976, Ganias 1990, Morri et al. 1999

#### Tubulipora

Lamarck, 1816

#### Tubulipora hemiphragmata

Harmelin, 1976

##### Notes

Castritsi-Catharios and Kiortis 1984, Castritsi-Catharios et al. 1985a, Castritsi-Catharios et al. 1985b, Castritsi-Catharios et al. 1986a, Castritsi-Catharios et al. 1986b, Ganias 1990

#### Tubulipora liliacea

(Pallas, 1766)

##### Notes

Castritsi-Catharios et al. 1985a, Ganias 1990

#### Tubulipora notomale

(Busk, 1875)

##### Notes

Ganias 1990

#### Tubulipora plumosa

Thompson in Harmer, 1898

##### Notes

Castritsi-Catharios and Ganias 1989, Ganias 1990, Morri et al. 1999

#### Tubulipora ziczac

Harmelin, 1976

##### Notes

Ganias 1990​

## Analysis

The bryozoan fauna of the Greek seas consists of 237 species, classified into 127 genera, 66 families, 3 orders, and 2 classes (Suppl. material 1). The vast majority belongs to the class Gymnolaemata, comprising 198 species (177 Cheilostomatida and 21 Ctenostomatida) and the remaining 39 species are Stenolaemata (all Cyclostomatida). The families with the highest number of species belong to Cheilostomatida, and are Phidoloporidae (16 species), Celleporidae (14 spp.), Bugulidae (12 spp.), Calloporidae (12 spp.), Smittinidae (11 spp.), Candidae (10 spp.), and Cribrilinidae (10 spp.). These families are the most speciose in the Mediterranean Sea as well (Rosso and Di Martino 2016). All other families include less than 10 species each.

All species included in the checklist are recognized in the World List of Bryozoa and the World Register of Marine Species, except for three of them. These are: *Idmidronea triforis* (Heller, 1967) and *I. coerulea* Harmelin, 1976, allocated within the genus *Idmidronea* Canu and Bassler, 1920 and not *Exidmonea* David, Mongereau and Pouyet, 1972; '*Microecia*' *occulta* (Harmelin, 1976), provisionally left in its former allocation because the characters of the species differ from those of the genus *Oncousoecia* Canu, 1918 that has been suggested for its allocation (although *Microecia* Canu, 1918 is recognized as a synonym of *Plagioecia* Canu, 1918). The taxon *Rhynchozoon* sp. 1 sensu Hayward, 1974 described from Chios Island, was retained in the checklist following extensive lists by Zabala and Maluquer 1988, Rosso et al. 2010, Rosso and Di Martino 2016 and taxonomic papers with description and illustrations of material from Tyrrhenian and Adriatic localities (Chimenz Gusso et al. 2014).

A number of taxa were omitted from the present checklist (Table 1) for different reasons: (1) taxa now recognized as species complexes and considered absent from the Mediterranean; further examination of specimens is needed to ascertain their correct identification; (2) species whose currently accepted geographic distribution does not include the Mediterranean Sea; the examination of the material with the Scanning Electron Microscope could possibly raise the number of non-indigenous species in the Mediterranean basin; (3) species known exclusively as fossils from Tertiary sediments of Europe, whose persistence in the Mediterranean Sea throughout the Messinian Salinity Crisis needs to be ascertained.

## Discussion

The bryozoan fauna of the Greek seas makes up 42.7% of the species, 59.9% of the genera and 71% of the families of the Mediterranean bryozoan fauna, specifically 41.7% of the Cheilostomatida, 36.8% of the Ctenostomatida and 52% of the Cyclostomatida species, based on the recent update by Rosso and Di Martino (2016). Despite the small number of sporadically distributed studies on this phylum in Greece, a considerable number of bryozoan species has been reported compared to adjacent eastern Mediterranean countries. A total of 185 species has been recorded from Turkey, 139 of which along the Aegean coasts (Koçak and Aydın Önen 2014) and 93 species from Lebanon (Harmelin et al. 2016), while 60 species were estimated to occur along the Mediterranean coasts of Israel (Sokolover et al. 2016). These numbers confirm the observed impoverishment of the bryozoan fauna from the western to the eastern basin (Harmelin et al. 2016).

Among the species reported from Greece, 12 are considered endemic to the eastern Mediterranean basin; these are the cheilostomes *Adeonella pallasii*, *Calyptotheca rugosa*, *Cellepora posidoniae*, *Hippopodina ambita*, *Hippoporidra picardi*, *Monoporella bouchardii*, *Plesiocleidochasma mediterraneum*, *Reteporellina delicatula*, *Retevirgula akdenizae*, *Smittipora disjuncta*, *Therenia rosei* and *Turbicellepora camera*. Of these, 5 species were first described from the island of Chios based on material collected by Hayward (i.e. *C. rugosa*, *H. ambita*, *R. delicatula*, *T. rosei*, and *T. camera*), *H. picardi* was described from the Gulf of Thessaloniki, while *R. akdenizae* was described from the Turkish coasts of the Aegean Sea. The cheilostome *Hippaliosina depressa*, which was described from the Aegean Sea, is considered more typical of the eastern Mediterranean basin, including the Sicily Strait, the western Ionian and south Adriatic seas (Harmelin et al. 2016 and references therein, AR personal observations). Nevertheless, this species was recorded in a few localities of western Corsica (Calvet 1902, Gautier 1962) and Sardinia (AR and E. Di Martino, personal observations) in the western Mediterranean.

Interestingly, three of the bryozoans recorded from Greece are typical of the North Atlantic (Hayward and Ryland 1998, Hayward and Ryland 1999) and have been rarely reported from the Mediterranean Sea. These are *Alderina imbellis* (Hincks, 1860), also reported from northern Catalonia (Madurell et al. 2013), *Escharina johnstoni* (Quelch, 1884), also reported from the western Ionian Sea (Rosso 1996a, Rosso 1996b, Rosso et al. 2014), and *Anarthropora monodon* (Busk, 1860).

The recent introduction of non-indigenous species, mostly lessepsian migrants, over the last decades, has considerably increased the number of bryozoans occurring in the Mediterranean Sea (Rosso and Di Martino 2016). Studies in the southeastern Mediterranean basin have brought to light a considerable number of NIBs (Harmelin 2014b, Gerovasileiou et al. 2016, Harmelin et al. 2016, Sokolover et al. 2016), mainly originated from the Red Sea/Indo-Pacific Ocean. Until today, 12 NIBs have been reported from the Greek seas, representing 5% of the bryozoan fauna of Greece and 20% of the Mediterranean NIB fauna; these are the ctenostomes *Amathia gracillima* and *A. verticillata* and the cheilostomes *Bugula neritina*, *Bugulina fulva*, *Crepidacantha poissonii*, *Crisularia plumosa*, *C. serrata*, *Exechonella antillea*, *Hippopodina feegeensis*, *Microporella coronata*, *Parasmittina raigii*, and *Scrupocellaria scruposa*.

Bryozoans from the Greek seas were reported from a variety of habitats, including soft sediments, seagrass leaves and rhizomes, macroalgae, coralligenous concretions and, to a smaller extent, from marine caves. Further research on bryozoan diversity in understudied habitats, typically species-rich in bryozoans (e.g. marine caves and coralligenous beds), is expected to increase our knowledge, possibly revealing additional new and non-indigenous species.

## Supplementary Material

Supplementary material 1 Checklist of Marine ﻿Bryozoa of Greece Data type: Taxonomic checklist
Brief description: Taxonomic checklist of Bryozoa known to occur in the Greek seas.
File: oo_104057.xls Vasilis Gerovasileiou, Antonietta Rosso

## Figures and Tables

**Table 1.** Questionable taxa reported from Greece not included in the present checklist.

**Species and authority as given in the source**	**First report from Greece**	**Remarks**
*Adeonella polystomella* (Reuss, 1847)	Harmelin (1969)	Omitted following Rosso and Novosel (2010).
*Cribrilina annulata* (Fabricius, 1780)	Castritsi-Catharios and Kiortis (1984)	Questionable (see Rosso and Di Martino 2016).
*Cribrilina punctata* (Hassall, 1841)	Castritsi-Catharios and Marcopoulou-Diacantoni (1983)	Possibly confused with *Collarina balzaci* (see Rosso and Di Martino 2016).
*Escharina porosa* (Smitt)	Hayward (1974)	Removed after the splitting of a previous complex of species (see Berning et al. 2008). Examination of material is needed.
*Hippodiplosia delicatula* Manzoni	Castritsi-Catharios et al. (1986)	Possibly an exclusively fossil species.
*Microecia suborbicularis* (Hincks)	Castritsi-Catharios et al. (1986)	The material for this species needs further examination (see Rosso et al. 2010).
*Monoporella fimbriata carinifera* Canu & Bassler, 1929 and *M. nodulifera* (Hincks)	Harmelin (1969), Hayward (1974)	Replaced by *M. bouchardii* following Harmelin (2014a).
*Parasmittina nitida* Verrill	Castritsi-Catharios et al. (1986)	Species restricted to the SW Atlantic (Winston and Maturo 2009).
*Pentapora foliacea*	Hayward (1974)	Species restricted to the North Atlantic (see Lombardi et al. 2010 and Rosso et al. 2010).
? *Rhyncozoon lobulatum* (Waters, 1879)	Harmelin (1969)	This species name is likely to refer to an aged portion of *R. digitatum* (see Zabala and Maluquer 1988).
*Schizomavella gardensis*	Castritsi-Catharios et al. (1986)	A fossil species described from the Burdigalian of France.
*Scrupocellaria reptans*	Hayward (1974)	Species restricted to the British Isles (see Rosso and Di Martino 2016 and references therein). All records from the Mediterranean (except for specimens from Alexandria attributed to *C. aegyptiaca*) need to be examined.
*Sertella scbuermanni* Jullien	Castritsi-Catharios et al. (1986)	This fossil species is reported only from its type locality (Miocene of west Germany).
*Tricellaria peachii* Osburn	Castritsi-Catharios et al. (1986)	Species restricted to the North Atlantic. Could be misidentified with *Tricellaria inopinata*.
*Tubulipora phalangea* (Couch, 1844)	Antoniadou and Chintiroglou (2005)	Species restricted to the Boreo-Arctic Atlantic Ocean (see Rosso and Di Martino 2016).
*Turbicellepora armata*	Castritsi-Catharios et al. (1985a), Castritsi-Catharios et al. (1985b)	This species is restricted to the North Atlantic.
*Turbicellepora redoutei* (Audouin, 1826)	Harmelin (1969)	Species seemingly restricted to the Indo-Pacific. It was recorded only once in the Mediterranean.
